# Immunoblotting validation of research antibodies generated against HS1-associated protein X-1 in the human neutrophil model cell line PLB-985.

**DOI:** 10.12688/f1000research.6516.2

**Published:** 2015-08-10

**Authors:** Peter Cavnar, Kristina Inman

**Affiliations:** 1 Department of Biology, University of West Florida, Pensacola, Florida, 32514, USA

**Keywords:** Hax1, neutrophil, PLB-985, tubulin

## Abstract

HS1-associated protein X-1 (Hax1) is a 35 kDa protein that is ubiquitously expressed. Hax1 is an anti-apoptotic protein with additional roles in cell motility, and autosomal recessive loss of Hax1 results in Kostmann syndrome, a form of severe congenital neutropenia. Because of the important role of Hax1 in neutrophils we demonstrate here validation of two commercially available research antibodies directed against human Hax1 in the human myeloid leukemia cell line PLB-985 cells. We show that both the mouse anti-Hax1 monoclonal IgG directed against amino acids 10-148 of Hax1 and a rabbit anti-Hax1 polyclonal IgG antibody directed against full-length Hax1 reliably and consistently detect Hax1 during immunoblotting of three different PLB-985 cell densities. Using shRNA mediated Hax1 knockdown, we demonstrate the specificity of both Hax1 antibodies. In addition, our results suggest that the rabbit anti-Hax1 polyclonal antibody provides a stronger intensity in detecting Hax1 protein, with detection in as few as 0.1 x 10 ^6^ cells in 6 total replicates we have performed.

## Introduction

HS1-associated protein X-1 (Hax1) is a 35 kDa protein consisting of 279 amino acids that is ubiquitously expressed ^1^. Hax1 has been demonstrated to be a negative regulator of apoptosis in many immune cell types ^2– 4^. Furthermore, Hax1 has been shown to have additional roles in regulating cell motility and adhesion ^5, 6^, and is overexpressed in many types of cancer ^7^. Patients with autosomal recessive mutations in the HAX1 gene have a form of severe congenital neutropenia called Kostmann syndrome. Severe congenital neutropenia is characterized by early recurrent bacterial infections and decreased neutrophil counts in the blood stream ^8^.

Because of the recent increase in Hax1 investigations, it is important to identify reliable antibodies directed against Hax1. Using the human neutrophil model cell line PLB-985 cells, which can be terminally differentiated into neutrophil-like cells after treatment with DMSO, we demonstrate the applicability and selectivity of two commercially available antibodies against Hax1. A mouse Hax1 monoclonal antibody (BD Biosciences) that is routinely used in publications investigating Hax1 ^5, 6, 9– 11^ directed against Hax1 amino acids 10–148, and a rabbit polyclonal antibody (Proteintech Group, Inc.) directed against the full length Hax1 protein ^6^.

## Materials and methods

### Reagent details

Details of all reagents used in the Western blotting procedures can be found in Table 1.

**Table 1.** Details of reagents used for immunoblotting.

Process	Reagent	Manufacturer	Catalogue Number	Concentration
6× Laemmli Protein Loading Buffer	Tris-HCL SDS Glycerol Bromophenol blue DL-Dithiothreitol	Fisher Scientific Fisher Scientific Fisher Scientific Sigma-Aldrich Sigma-Aldrich	Tris base BP152 BP166-100 G33-1 B0126 D0632	375mM 9% 50% 0.03% 0.6M
Protein blotting	0.45μm nitrocellulose pure transfer membrane	Santa Cruz Biotechnology	Sc-201705	
SDS-PAGE Transfer Buffer	1× Tris-Gylcine Electroblotting buffer Methanol	National Diagnostics Fisher Scientific	EC-800 A412-4	1× (25mM Tris-HCL, 192mM glycine) 20% v/v
Wash Buffer, blocking buffer, and Antibody Diluent	Tween-Tris/Saline (T-TS)	Fisher Scientific	Tris base BP152 NaCl S271 Tween-20 BP337	50mM Tris 150mM NaCl 1% Tween -20
Blocking	Bovine Serum Albumin, heat shock fraction	Sigma-Aldrich	A9647	5% in T-TS
Pre-Immune Serum Incubation ( Figure 5A)	Rabbit pre-immune serum Mouse pre-immune serum	Sigma-Aldrich Sigma-Aldrich	R9133 M5905	1:1000 1:1000

### Antibody details

Anti-tubulin (beta-) is a mouse monoclonal IgG1 [E7 was deposited to the DSHB by Klymkowsky, Michael (DSHB Hybridoma Product E7)] and was used as a loading control for all Western blots at a dilution of 1:1000 resulting in a final concentration of 45 ng/mL. Rabbit anti-Hax1 (Proteintech Group, Inc, Table 2) is a polyclonal antibody generated to full length *Homo sapiens* Hax1. The lot number used was 1, and a dilution of 1:1000 was used for all Western blots resulting in a final concentration of rabbit anti-Hax1 of 230 ng/mL. Mouse anti-Hax1 (BD Biosciences) is a mouse monoclonal IgG1 raised against *Homo sapiens* Hax1 amino acids 10–148. The lot number used was 3266979, and a dilution of 1:1000 was used for all Western blots resulting in a final concentration of 250 ng/mL. Goat anti-rabbit IgG IRDye 680LT and Goat anti-mouse IgG IRDye 800CW (Li-Cor Biosciences, Table 2) were used at a dilution of 1:40,000 (25 ng/mL).

**Table 2.** Details of Primary and Secondary Antibodies.

Antibody	Manufacturer	Catalogue number	RRID	Concentration used
Tubulin (beta-)	Developmental Studies Hybridoma Bank	E7-s	RRID:AB_528499	45 ng/mL
Hax1	BD Biosciences	610824	RRID:AB_398143	250 ng/mL
Hax1	Proteintech Group, Inc.	11266-1-AP	RRID:AB_2263720	230 ng/mL
Goat anti-Rabbit IgG IRDye 680LT	Li-Cor Biosciences	926-32221	RRID:AB_621841	25 ng/mL
Goat anti-Mouse IgG IRDye 800CW	Li-Cor Biosciences	827-08364	RRID:AB_10793856	25 ng/mL

### Cell culture

PLB-985 cells were maintained in RPMI 1640 (Mediatech, Inc.) supplemented with 10% fetal bovine serum, 60 μg/mL penicillin, and 100 μg/mL streptomycin (Mediatech, Inc.) at a concentration of 0.1–1 × 10 ^6^ cells/mL. To differentiate PLB-985 cells into “neutrophil-like” cells 1.25% DMSO (Fisher Scientific) was added to 2 × 10 ^5^ cells/mL for 6 days. Lentiviral Hax1 shRNA targets were purchased from Open Biosystems. Targets used; Hax1 shRNA (5'-ACAGACACTTCGGGACTCAAT-3') and control shRNA (5'-TGTCTCCGAACGTGTCACGTT-3'). HEK293-FT cells were grown to 70% confluency in a 10cm tissue culture dish for each lentiviral target and transfected using 6μg Hax1, 0.6μg vesicular stomatitis virus (VSV)-G, and 5.4μg cytomegalovirus (CMV) 8.9.1. 72 hour viral supernatant was collected and concentrated using Lenti-X concentrator (Clontech, Inc.) following the manufacturer’s instructions. 1 × 10 ^6^ PLB-985 cells were infected with viral supernatant for 3 days in the presence of polybrene (4 μg/mL, Santa Cruz Biotechnology). Stable cell lines were generated with puromycin (1 μg/mL, Sigma Aldrich) selection.

### Immunoblot analysis

- Differentiated PLB-985 cells were counted and 0.1 × 10 ^6^, 0.5 × 10 ^6^, and 1 × 10 ^6^ cells were pelleted by centrifugation.
- Cells were lysed in Triton X-100 lysis buffer with protease inhibitors (25 mM HEPES, pH 7.5, 150 mM NaCl _2_, 1% TX-100, 10 mM MgCl _2_, 1 mM EDTA, 10% glycerol, 1 μg/mL pepstatin A, 2 μg/mL aprotinin, 1 μg/mL leupeptin) on ice for 10 minutes and clarified by centrifugation.
- Cellular lysate was then removed and added to 6× Laemmli sample buffer, boiled at 90°C for 5 minutes, and run on 10% SDS-PAGE gels.
- Proteins were then transferred to 0.45μm nitrocellulose membranes (Santa Cruz Biotechnology) at 400mA for 1 hour at 4°C.
- Following transfer, the membrane was blocked in 5% BSA in 1× T-TS for 1 hour at room temperature with gentle rocking.
- Membranes were then probed with mouse anti-tubulin [(beta-) (45 ng/mL)], and either mouse anti-Hax1 (BD Biosciences, 250 ng/mL) or rabbit anti-Hax1 (Proteintech Group, Inc., 230 ng/mL) at room temperature for 1 hour.
- After primary antibody incubation the membranes were washed 3 × 5 minutes with 1× Tris-HCL/NaCl saline buffer (1× T-TS), see Table 1.
- The membranes were incubated with goat anti-rabbit IgG IRDye 680LT and goat anti-mouse IgG IRDye 800CW (Li-Cor Biosciences, 25 ng/mL) at room temperature for 1 hour.
- After secondary antibody incubation the membranes were washed 3 × 5 minutes with 1× T-TS.
- Blots were imaged with an infrared imaging system (Odysssey Fc; Li-Cor Biosciences) using a 2-minute exposure time.

## Results

**Figure 1.**
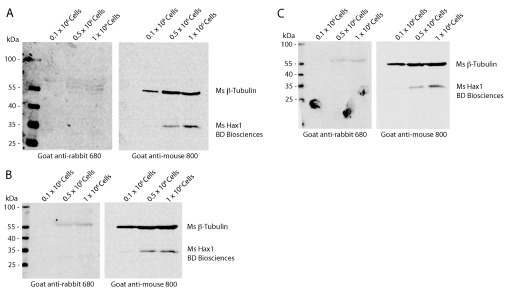
Detection of Hax1 in differentiated PLB-985 cells using a mouse anti-Hax1 antibody. Western blot analysis of differentiated PLB-985 cell lysates from 0.1 × 10 ^6^, 0.5 × 10 ^6^, and 1 × 10 ^6^ cells from three independent replicates. Mouse anti-tubulin (beta-) is used as a loading control and can be seen present at a relative mobility of 55 kDa in the goat anti-mouse 800 channel. Mouse anti-Hax1 detects a band with a relative mobility of 35 kDa as predicted. Hax1 can be detected in densities of 0.5 × 10 ^6^ and 1 × 10 ^6^ cells.

**Figure 2.**
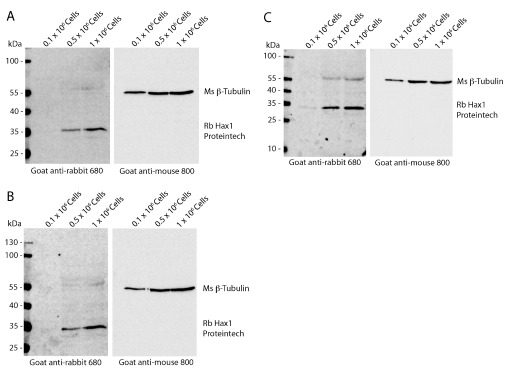
Detection of Hax1 in differentiated PLB-985 cells using a rabbit anti-Hax1 antibody. Western blot analysis of differentiated PLB-985 cell lysates from 0.1 × 10 ^6^, 0.5 × 10 ^6^, and 1 × 10 ^6^ cells from three independent replicates. Mouse anti-tubulin (beta-) is used as a loading control and can be seen present at a relative mobility of 55 kDa. Rabbit anti-Hax1 detects a band at a relative mobility of 35 kDa as predicted. Hax1 can be detected in densities as low as 0.1 × 10 ^6^ cells ( **C**).

**Figure 3.**
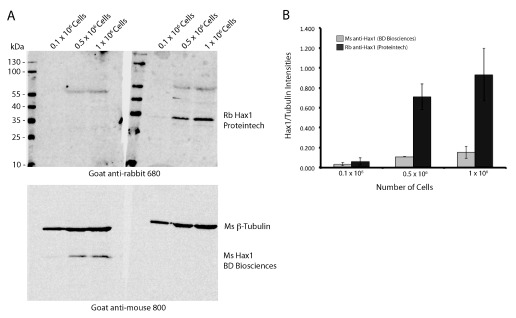
Comparison of mouse and rabbit anti-Hax1 antibodies in differentiated PLB-985 cells. ( **A**) Western blot analysis of differentiated PLB-985 cell lysates from 0.1 × 10 ^6^, 0.5 × 10 ^6^, and 1 × 10 ^6^ cells comparing mouse and rabbit anti-Hax1 antibodies. Lysates from the same cell extractions were run on a single SDS-PAGE gel and blotted onto a single nitrocellulose membrane. After transfer, the membrane was divided and probed with either mouse anti-Hax1 or rabbit anti-Hax1. The membranes were imaged simultaneously. ( **B**) Quantification of the ratios of Hax1 and tubulin band intensities from three independent blots were measured and plotted. Error bars indicate standard deviation.

**Figure 4.**
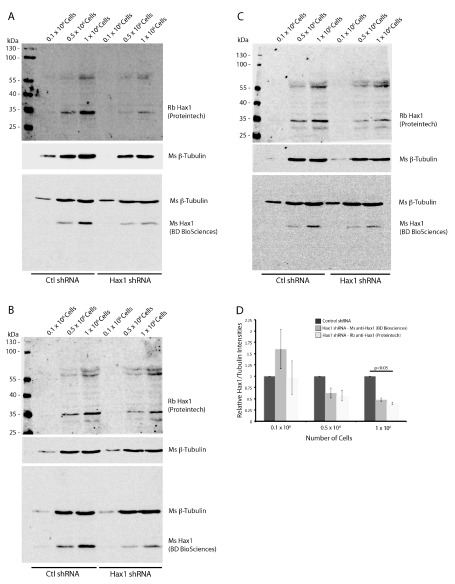
Detection of Hax1 in Control shRNA and Hax1 shRNA expressing differentiated PLB-985 cells using mouse and rabbit anti-Hax1 antibodies. ( **A**– **C**) Western blot analysis of differentiated PLB-985 cell lysates from 0.1 × 10 ^6^, 0.5 × 10 ^6^, and 1 × 10 ^6^ cells expressing either control shRNA or Hax1 shRNA from three independent replicates. Mouse anti-tubulin (beta-) is used as a loading control and can be seen present at a relative mobility of 55 kDa. Both mouse and rabbit anti-Hax1 detects a band at a relative mobility of 35 kDa as predicted. ( **D**) Quantification of the band intensities of tubulin and Hax1 relative to control shRNA from three independent Western blots. Error bars indicate standard error of the mean. p values were calculated using paired t-test to assess significance relative to control shRNA.

To determine the reproducibility and sensitivity of the mouse and rabbit anti-Hax1 antibodies on the PLB-985 cells, we performed Western blot analysis using three separate cell densities, 0.1 × 10 ^6^, 0.5 × 10 ^6^, and 1 × 10 ^6^ cells. In our research using the PLB-985 cell system, we routinely use 1 × 10 ^6^ – 10 × 10 ^6^ cells in a Western blot. Using beta-tubulin as a loading control our Western blots illustrate an increasing protein concentration in the three samples as would be expected with increasing cell densities. We found that the mouse anti-Hax1 antibody (BD Biosciences) is visible as low as 0.5 × 10 ^6^ cells, binding to a protein band at the expected Hax1 size with a relative mobility of 35 kDa ( Figure 1). In six different experiments ( Figure 1 and Figure 4) we found inconsistency in protein detection with the Ms anti-Hax1 antibody. In all blots Hax1 was visible, however with varying degrees of intensity. Conversely, when the rabbit anti-Hax1 antibody (Proteintech Group, Inc.) was used, the antibody gave consistent and robust detection ( Figure 2 and Figure 4). In some cases, Hax1 can be detected in as low as 0.1 × 10 ^6^ cells using the rabbit anti-Hax1 antibody ( Figure 2C). We do not believe the difference between the two antibodies is due to variations in the cell extract or imaging software because when the same cell extract is immunoblotted on two different blots and scanned simultaneously the difference in sensitivity can be observed ( Figure 3A). Using the Odyssey imaging system (Li-Cor Biosciences) to measure the intensity of each band, we calculated the intensity ratio of Hax1 relative to the tubulin loading control from three independent blots for each antibody ( Figure 3B). In both blots the levels of tubulin are similar, however it is evident that the rabbit anti-Hax1 antibody exhibits a stronger signal compared to the mouse monoclonal antibody. Nevertheless, it should be noted that both antibodies reliably detect Hax1 in differentiated PLB-985 cells.

**Figure 5.**
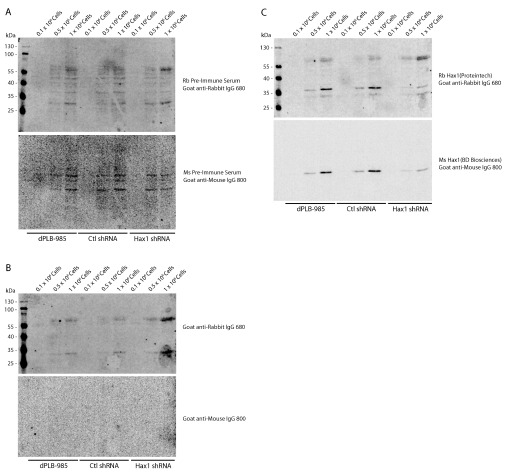
Goat anti-Rabbit IgG secondary antibody only background detection of differentiated PLB-985 cell lysates. ( **A**) Western blot analysis using rabbit and mouse pre-immune serum from 0.1 × 10 ^6^, 0.5 × 10 ^6^, and 1 × 10 ^6^ differentiated PLB-985, control shRNA, and Hax1 shRNA cells. ( **B**) Western blot analysis using goat anti-rabbit IgG 680LT only on cell lysates from 0.1 × 10 ^6^, 0.5 × 10 ^6^, and 1 × 10 ^6^ differentiated PLB-985, control shRNA and Hax1 shRNA expressing PLB-985 cells. Two predominant background bands can be observed at a relative mobility of 60 and 70 kDa, and one band around 30 kDa. These background bands can also be seen in Figure 1, Figure 2, and Figure 4. ( **C**) Subsequent incubation with rabbit and mouse anti-Hax1 from Western blots shown in **B** demonstrate the appearance of the Hax1 band at the predicted 35 kDa size.

To demonstrate the specificity of both Hax1 antibodies we generated stably-expressing control shRNA and Hax1 shRNA PLB-985 cells ( Figure 4). As described previously using the mouse anti-Hax1 antibody the control shRNA cells show inconsistent staining intensity, however in these samples the mouse anti-Hax1 antibody is more robust than in the wild-type PLB-985 cells. Both the mouse anti-Hax1 and rabbit anti-Hax1 antibodies show reduced detection in the Hax1-deficient PLB-985 cells. Quantification of the level of Hax1 knockdown is consistent using the two antibodies at 1 x 10 ^6^ cells. This demonstrates that the antibodies are highly specific for Hax1. In many of the experiments we observed additional background bands in the rabbit 680nm channel. To determine the source of these background bands rabbit and mouse pre-immune serum were tested ( Figure 5A). Our results show a unique background pattern using the pre-immune serum that we do not observe on the Hax1 blots. We next performed a Western blot on cells using only the rabbit and mouse secondary antibodies ( Figure 5B). The mouse channel does not display any significant background, however the rabbit secondary antibodies shows a background staining that we observe in the previous blots as well. These blots were then subsequently probed with the rabbit and mouse Hax1 antibodies ( Figure 5C). Comparison of the secondary only blot before and after Hax1 antibody incubation demonstrates that the Hax1 antibodies are highly specific and the background we are observing can be attributed to the goat anti-rabbit IgG secondary antibody.

Raw data for Figure 3 quantification Comparison of mouse and rabbit anti-Hax1 antibody band intensities in differentiated PLB-985 cells. Quantification of the band intensities from three independent Western blots was measured and the ratios of Hax1 to tubulin were plotted.

Raw data for Figure 4 quantification Detection and quantification of Hax1 in control shRNA and Hax1 shRNA expressing PLB-985 cells. Quantification of the band intensities was measured and the ratios of Hax1 to tubulin were plotted relative to the control shRNA ratios for each cell density assayed. An average, standard deviation, and standard error of the mean were calculated for each cell density and each antibody used from three independent replicates.

## Conclusion

Here we show validation and comparison results of two commercially available antibodies generated against HS1-associated protein X-1 (Hax1), an anti-apoptotic protein that has a multi-factorial role in regulating cell proliferation and differentiation, cell motility, and cancer. Homozygous loss-of-function of Hax1 results in severe congenital neutropenia, a life threatening loss of circulating neutrophils in the blood stream. Studying the function of Hax1 in primary neutrophils and the neutrophil model cell line PLB-985 will help elucidate the disease pathogenesis of neutropenia syndromes. We demonstrate that mouse anti-Hax1 (BD Biosciences) and rabbit anti-Hax1 (Proteintech Group, Inc.) are both specific for Hax1. Furthermore we show that as little as 0.5 × 10 ^6^ differentiated PLB-985 cells can be used to reliably detect Hax1 expression with both of the antibodies. We have evidence that the rabbit anti-Hax1 (Proteintech Group Inc.) results in a more robust and consistent detection of Hax1, likely due to the polyclonal nature of the antibody. Finally, lentiviral knockdown of endogenous Hax1 expression results in loss of Hax1 detection by both mouse anti-Hax1 and rabbit anti-Hax1 demonstrating the specificity of each antibody. In our quantification of Hax1 knockdown we observed variation when the cell densities were low, with 1 × 10 ^6^ cells giving us the most reliable quantification. In our experiments we observed background bands that we attributed to the goat anti-rabbit 680nm secondary antibody. Therefore we are confident that these antibodies are very specific.

In conclusion we recommend the use of either mouse or rabbit anti-Hax1 antibodies shown here for studies using the PLB-985 cells as a neutrophil model cell line. It is our conclusion that a minimum cell density of 0.5 × 10 ^6^ neutrophils should be used as a starting point for immunoblotting of Hax1, with greater than or equal to 1 × 10 ^6^ cells being optimal.

## Data availability

*F1000Research*: Dataset 1. Raw data for Figure 3 quantification., 10.5256/f1000research.6516.d99343 ^12^

*F1000Research*: Dataset 2. Raw data for Figure 4 quantification., 10.5256/f1000research.6516.d99344 ^13^
