# Supplementary material for: Immunoblotting validation of research antibodies generated against HS1-associated protein X-1 in the human neutrophil model cell line PLB-985
Source: F1000Res. 2015 Aug 10;4:148. Originally published 2015 Jun 10. [Version 2] doi: 10.12688/f1000research.6516.2 (PMC4893944; doi:10.12688/f1000research.6516.2)
Supplement: Peer review history

# Peer review: Referee response for version 1

**DOI:** 10.5256/f1000research.6992.r9247

**Published:** 2015-07-08

**Authors:** Lawrence L. LeClaire III

**Affiliations:** 1 Department of Biochemistry and Molecular Biology, University of South Alabama, Mobile, AL, USA

Kristina and Cavnar present data to validate two commercial antibodies for HSA-associated protein X-1 in a human neutrophil cell line. The authors describe the conditions for the experiments as well as provide sufficient information/protocols for reader about the conditions used in that assays. In addition, the authors present the entire blots and describe the secondary bands that were detected.  Only minor changes in the text should be made (below).

1. The commercial source of the antibody should be listed in the abstract. It currently gives the impression that the antibodies used in the study were generated by the authors.
2. The electrophoretic transfer conditions should be listed. Transfer buffer contents (%MeOH) as well as the model of transfer equipment (dry, semi-dry) will cause variability in protein transfer/antibody detection.
3. Did the authors attempt to vary blocking conditions (milk, commercial blocking agents)?
4. Is purified HAX1 available to use as a positive control or to build a standard curve for quantifying total HAX1 in the samples? Not necessary for this publication but would be extremely useful. Figure 3B suggests that detection is close to linear (especially with LiCor technology).
5. Authors should denote the size of bands on an SDS-PAGE as "relative mobility." For example, a band with a relative mobility of 32 kDa was detected...
6. In Figure 3, change "After transfer, the membrane was cut.." To "the membrane was divided"
7. Are the authors certain that the band detected by the secondary antibody is the lower of the two bands in the experiments? A single lane could be divided and one side probed with primary and the other with only secondary to confirm.

I have read this submission. I believe that I have an appropriate level of expertise to confirm that it is of an acceptable scientific standard.

# Response

**Published:** 2015-08-03

**Authors:** Peter Cavnar

**Affiliations:** University of West Florida, USA

Thank you for your kind review. I have revised and submitted a new version of this manuscript. Per your questions I will address each one:

1. I have included the commercial availability of the antibodies in the abstract, introduction, and discussion.
2. The transfer buffer conditions have now been included in Table 1.
3. We have tried blocking with non-fat dry milk and do not find any differences in the selectivity of the Hax1 antibodies, however we did get considerable background from the milk in the goat anti-rabbit 680 channel. We have tested this with a blank membrane. Therefore we will traditionally use 5% BSA to block.
4. We do have bacterial constructs that could be used to purify Hax1. Naturally that was not the purpose of this study, but I concur that these antibodies do behave quite linearly.
5. Thank you for this suggestion, we have included all sizes as a relative mobility in our results and figure legends.
6. We have edited figure 3 legend to reflect this.
7. We have revised figure 5 which demonstrates that the background band that has a relative mobility of 32 kDa is from the secondary antibody.

# Peer review: Referee response for version 1

**DOI:** 10.5256/f1000research.6992.r9249

**Published:** 2015-07-06

**Authors:** Mautusi Mitra

**Affiliations:** 1 Department of Biology, University of West Georgia, Carrollton, GA, USA

The paper titled “"Immunoblotting validation of research antibodies generated against HS1-associated protein X-1 in the human neutrophil model cell line PLB-985." by Kristina and Cavnar focuses on the validation of two commercial antibodies generated against the human Hax1 protein. The experiments are in most part well designed. The research approaches, methods and the reagents used for the experiments are well described. The researchers have shown the entire Western blot and have also used Hax1-deficient PLB-985 cell lines to demonstrate the specificity of the two Hax1 antibodies. The data presented in the article strongly indicate that both the antibodies, especially the rabbit anti-Hax1 polyclonal antibody, can be used for specific detection of the human Hax1 protein and also shows the minimum cell density of neutrophils that can be used for immunoblotting experiments.

The manuscript is suitable for indexing, provided the authors address few issues, which are described below:

1. It is not clear if the antibodies were purchased commercially or were generated by the research laboratory with the help of a commercial company. This should be clarified.
2. Hax1 interacts with the polycystic kidney disease protein PKD2, located in the ER membrane/plasma membrane protein. Have the authors checked the efficiency of protein solubilization in their experiments? Have the authors done western analyses on a blot which has proteins transferred from a SDS-PAGE gel that has the intact stacking gel? If the solubilization is incomplete, insoluble proteins will be detected by the respective antibody in the stacking wells in the blot. This experiment can help to confirm the minimum cell density of neutrophils to be used for immunoblotting and might help to improve the sensitivity of the Hax1 detection by these antibodies.
3. The authors should specify the detailed composition of the Laemmli sample buffer as the composition of this buffer varies slightly from lab to lab. Some research labs add 2M-4M urea in the Laemmli buffer for complete solubilization of membrane bound- hydrophobic proteins that have a tendency to precipitate in SDS when heated at 95°C. If urea plus SDS are used for sample solubilization, samples are incubated at room temperature for 30 minutes [as heating causes urea to break down].
4. Have the authors tried immunoblotting with the pre-immune serum to make sure that these antibodies do not have any non-specific interactions? The authors should make this clear in the manuscript.
5. The authors should clearly state the duration of the secondary antibody incubation which is missing in the manuscript.
6. The authors state that the background protein bands are due to the goat anti- rabbit IgG secondary antibody (Figure 5). But there are background bands with the goat anti-mouse IgG secondary antibody (Figure 5). Have the authors performed the same experiment with the goat-anti-mouse secondary antibody? Is the background due to the cell line used in the experiment or due to the specific batch of the antibody? It would be nice to discuss this issue in the discussion section of the manuscript.
7. No explanation was given for the observation why the control shRNA cells show more intense staining intensity than the wild type cells. This should be addressed in the discussion.
8. Figure 3B does not clearly state how many replicates were used for the quantification of the band intensities.
9. The authors should quantify the Hax1 knockdown in the Hax1 shRNA cell line (Figure 4). Without quantification, it is hard to accurately distinguish the specific Hax1 reduction from the background non-specific weak binding of antibodies.

I have read this submission. I believe that I have an appropriate level of expertise to confirm that it is of an acceptable scientific standard.

# Response

**Published:** 2015-08-03

**Authors:** Peter Cavnar

**Affiliations:** University of West Florida, USA

Thank you for your kind review of our manuscript. Below I have addressed your comments:

1. We have clarified in the abstract, introduction, and discussion that these antibodies are commercially available.
2. We have performed these experiments using gels that have not had the stacking gel removed and we do not observe any noticeable signal in the stacking gel. Furthermore, Gallagher *et al. *use a 1% TX-100 lysis buffer in their experiments similar to our lysis conditions. It would be interesting to purify the nuclear extract and compare to the cytosolic fractions to determine this further.
3. The composition of the Laemmli sample buffer has now been added to Table 1.
4. Thank you for this suggestion. Pre-immune sera experiments have been added to figure 5. In short we find that there is increased background using the pre-immune sera that we don't typically see. This demonstrates that the commercially available antibodies are relatively clean after the purification process.
5. The 1 hour incubation of the secondary antibody has been added to the manuscript.
6. The apparent background bands noticed in the mouse channel was a mistake on our part. For many of the figures the rabbit and mouse channels were overlays. This has been fully corrected and every figure now contains separate blots for the mouse and rabbit channels. As can be seen the background is only observed in the rabbit channel.
7. We have repeated these experiments and on average we do not see any significant increase in band intensities between the wild-type PLB-985 cells and the control shRNA cells (see Figure 5C). This could have been variation due to loading in the past figures. We hope the new figures are more clear in this regard.
8. Figure 3B is quantification from three independent replicates. This has been clarified in the figure legend, and the included dataset 1 contains the raw numerical information.
9. This has been completed. Quantifying the knockdown from the 0.1 x 10^6 and 0.5 x 10^6 cell samples was highly variable due to the low level of protein available, however at the 1 x 10^6 cell density quantification was reliable and both antibodies showed a relatively equal level of knockdown.

# Peer review: Referee response for version 1

**DOI:** 10.5256/f1000research.6992.r8986

**Published:** 2015-06-18

**Authors:** Andrew Chalmers

**Affiliations:** 1 Department of Biology and Biochemistry, University of Bath, Bath, UK

The work presented by Kristina and Cavnar presents data validating two commercial antibodies raised against Hax1. Their experimental approach is well explained and there is a good description of the methods and reagents used. The data is also convincing and well presented, I particularly liked the fact that complete blots are shown and knockdown used to show specificity.  Overall, it is a very strong example of an antibody validation paper.

I believe the manuscript is suitable for indexing subject to a few fairly minor changes, which are detailed below:

1. I would make it clear that the antibodies are commercially available in the abstract.
2. It would be worth double checking that details are provided for all of the western blotting reagents and methods. There is plenty of evidence that small changes can alter antibody behaviour and it really helps those trying to reproduce the work. One thing I noticed is that I don’t think the contents of Laemmli loading buffer are spelt out, does it have reducing agent in it? Another example is how long were secondaries incubated for?
3. Figure 3- Is the quantification based on one or more experiments- this should be clear in the legend.
4. Dataset 1- should be “plotted” and not “graphed”.
5. I was interested in the background which appears to be caused by the secondary antibodies. The authors state it is due to the anti-rabbit antibody, but it seems there is at least some with the anti-mouse staining? I would be interested if this is common with this cell line or is it this set of secondaries- I think this would be worth briefly mentioning in the discussion.
6.  The authors should present the quantification of the knockdown (figure 4). If both antibodies are specific then you would predict that they will show roughly similar levels of reduction in signal. This would suggest that the remaining signal is caused by a lack of knockdown rather than low levels of non-specific staining.

I have read this submission. I believe that I have an appropriate level of expertise to confirm that it is of an acceptable scientific standard.

# Response

**Published:** 2015-08-03

**Authors:** Peter Cavnar

**Affiliations:** University of West Florida, USA

Thank you for the kind review of our manuscript. We have addressed your comments below:

1. Commercial availability of the antibodies has now been included in the abstract, introduction, and discussion.
2. We have now included the composition of the Laemmli loading buffer and also our transfer buffer composition to Table 1.
3. Figure 3 includes quantification from three independent experiments.
4. All references to datasets and graphs have been changed to "plotted".
5. This was a mistake on our part in our first submission the two channels were overlays. This has been corrected and all blots have been separated into their subsequent mouse and rabbit channels. We hope it is clear now that the background bands we see are due to the goat anti-rabbit 680 secondary antibody with no detectable background in the mouse channel.
6. This was a great suggestion and has been completed. The quantification is highly variable at low cell densities, however at 1 x 10^6 cells the level of the Hax1 KD is relatively constant and significant using the mouse or rabbit Hax1 antibodies. This demonstrates the specificity of the Hax1 antibodies used in this study.
